# Stage-dependent analysis of IL-6, TGF-β, osteocalcin, and sRANKL in the synovial fluid of dogs with osteoarthritis

**DOI:** 10.1038/s41598-026-56559-6

**Published:** 2026-06-09

**Authors:** Stephan Neumann, Hanna Diekmann, Sarah Lauenstein

**Affiliations:** Institute of Veterinary Medicine, University of Göttingen, Burckhardtweg 2, 37077 Göttingen, Germany

**Keywords:** Pathogenesis, Biomarkers

## Abstract

Osteoarthritis (OA) is traditionally considered a degenerative joint disease. However, morphological similarities between OA progression and fracture healing suggest that OA may be a dysregulated bone repair process. This study investigated the morphological and biochemical parallels between OA and bone healing in dogs following cranial cruciate ligament rupture. To differentiate between biochemical profiles at different stages of the disease, a structured OA scoring system was developed based on radiographic and additional intraoperative findings. Synovial fluid samples from the OA cohort were analysed for IL-6, TGF-β, osteocalcin and sRANKL. IL-6 concentrations decreased significantly with advancing OA severity (*p* = 0.0068), suggesting a shift from inflammatory to reparative mechanisms. TGF-β, osteocalcin and sRANKL demonstrated stage-dependent trends consistent with osteogenic and remodelling activity. However, these associations did not reach statistical significance. The combined morphological and biomarker findings suggest that OA progression exhibits features similar to the phases of bone healing, which are characterised by dynamic interactions between inflammation, resorption, and bone formation. Nevertheless, the predominantly non-significant biomarker correlations highlight the need for larger sample sizes and longitudinal studies to confirm these observations and further clarify causal mechanisms. These results support the concept that OA may represent a chronic but dysregulated adaptive repair response to joint damage, rather than a purely degenerative process.

**Supplementary Information:**

The online version contains supplementary material available at 10.1038/s41598-026-56559-6.

## Introduction

Osteoarthritis (OA) is one of the most common degenerative joint diseases and represents a significant health problem. The disease is characterized by the progressive degradation of articular cartilage, structural changes in the subchondral bone, synovial inflammation and the formation of osteophytes^1–5^. In the final stage of OA, a complete remodeling of the joint structure can occur, which is referred to as ankylosis. The joint is replaced by compact bone tissue, which results in a complete restriction of movement^6–8^.

Bone healing, on the other hand, is a highly orchestrated biological process that takes place after a fracture with the aim of restoring the original function and stability of the bone^9–12^. This process involves several phases, including an initial inflammatory reaction, the formation of soft and subsequent hard callus and final bone remodeling^13–15^. When bone healing is disrupted, as occurs in pseudarthrosis, the restoration of bone continuity remains incomplete, resulting in an unstable, joint-like structure^16–18^.

The phenomena of ankylosis, as a consequence of advanced joint remodeling, and pseudarthrosis, as a consequence of impaired bone healing, suggest pathogenetic parallels. These observations lead to the hypothesis that OA can be interpreted as a kind of “bone healing in the wrong place^19–22^. Molecular mechanisms of bone healing, which normally contribute to the restoration of bone integrity, could be dysregulated and misdirected in OA^23–25^.

The process of bone healing is controlled by numerous cytokines and growth factors. Certain molecules are representative of specific mechanisms.

IL-6, for example, is a pro-inflammatory cytokine that plays a key role in the initial inflammatory phase of bone healing. It is produced by various cell types such as macrophages, T cells and fibroblasts and promotes osteoclastogenesis by inducing the expression of RANKL (Receptor Activator of Nuclear Factor κB Ligand)^26–28^. These mechanisms are crucial for the degradation of damaged bone structures and allow the repair and reconstruction of healthy bone tissue^29,30^.

In the context of osteoarthritis, IL-6 expression leads to inflammation, which can induce progressive cartilage degradation^31,32^.

TGF-β (transforming growth factor beta) is a multifunctional cytokine that plays a central role in both the repair and remodeling phases of bone healing. It promotes the differentiation of mesenchymal stem cells into osteoblasts and supports the synthesis of bone-forming matrix proteins such as collagen and osteocalcin^33–35^. At the same time, TGF-β regulates osteoclast activity by suppressing the production of RANKL, thus maintaining the balance between bone resorption and formation^36^. In osteoarthritis, increased synthesis of TGF-β induces fibrosis and ossification of the joint tissue^37,38^.

sRANKL is an essential molecule for the regulation of osteoclast formation and activity. It binds to the sRANK receptor on the surface of osteoclast precursor cells and initiates their differentiation into mature osteoclasts^39–41^. During bone healing, sRANKL is involved in the resorption of damaged bone areas, a process that is essential for the subsequent formation of stable bone structures^42^.

In osteoarthritis, the regulation of sRANKL and RANK is often disturbed, which leads to excessive bone resorption. This contributes to the destruction of the joint structures^43^.

Osteocalcin is a protein that is synthesized by mature osteoblasts during the bone formation phase. It binds to hydroxyapatite and plays a crucial role in the mineralization of the bone matrix^44–46^. In addition, osteocalcin has systemic effects, including influencing glucose metabolism and insulin sensitivity^47^. Its concentration reflects the activity of osteoblasts and is often used as a marker for the state of bone healing^48–50^.

Although numerous studies have investigated inflammatory cytokines and bone remodelling markers in human osteoarthritis, data on canine osteoarthritis (OA) remains comparatively limited. Most research in dogs has focused on inflammatory mediators associated with synovitis and cartilage degradation. Increased concentrations of cytokines such as IL-1β, TNF-α and IL-6 have been reported in canine synovial fluid during the early stages of OA, which supports the idea that inflammation plays a role in the initiation of the disease^51,52^.

This study aims to systematically investigate the morphological and biochemical parallels between osteoarthritis and bone healing. Using a specific OA scoring system, the severity and progression of the disease will be analyzed. In addition, the molecular analysis focuses on the biomarkers IL-6, TGF-β, sRANKL and osteocalcin, which have central functions in the phases of bone healing. These markers will be examined in the context of OA for their role in the pathological processes. The aim is to decipher the mechanisms underlying the hypothesis of “bone healing in the wrong place”^53–55^.

## Results

### Morphological changes and OA assessment

Radiologic and histopathologic analysis of the joint structures revealed progressive morphologic changes that correlated with increasing OA scores.

### OA grade 1

In animals with OA score 1, histologic examination revealed primarily inflammatory changes in the synovial membrane. Mild lymphoplasmacytic synovitis, minimal synovial hyperplasia and discrete fibroblast infiltration were characteristic. The joint capsule showed edema, which was primarily attributable to inflammatory processes, while structural fibrosis was not significantly detectable. Pathological changes in the articular cartilage were not observed at this stage. Radiographic findings at this stage were roughness on predisposed surfaces such as the condyles, patella and the facies poplitea.

### OA grade 2

**Fig. 1:**
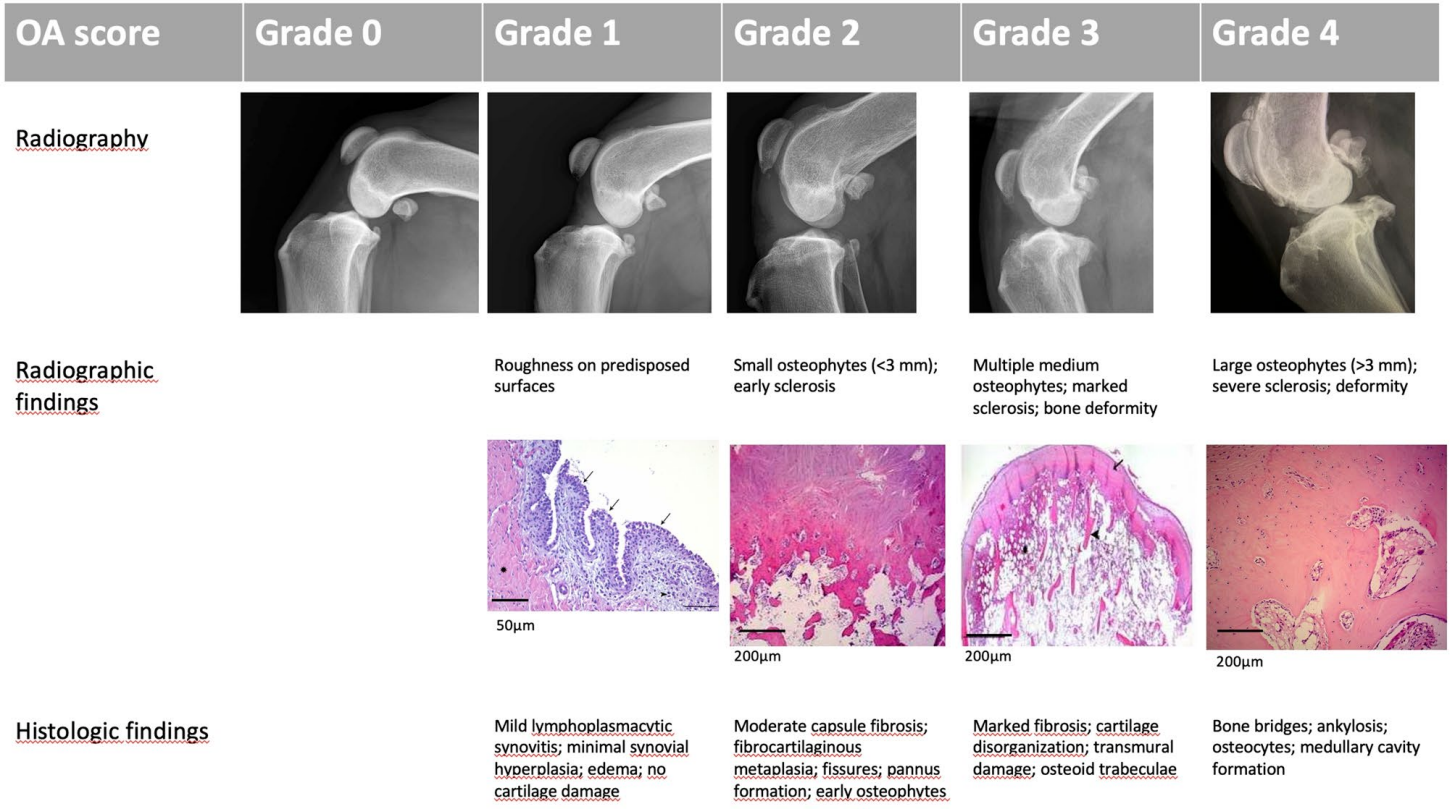
Histologic and radiologic findings in dogs with OA score 1 to 4. With description of radigraphic and histological findings. Grade 1: Joint capsule (star). The synoviocytes show mild to moderate villous hyperplasia with villus-like protrusions of the synovial membrane (arrows). Mild inflammatory cell infiltrates are found in the subsynovial interstitium (arrowhead). Grade 2: Chondroblasts, chondrocytes, including osteoblasts and osteoclasts deep within mature bone. Grade 3: Overview Osteophyte; cartilage layer (arrow) with loosened to fibrillar matrix and very narrow proliferation zone (star) as well as some adhering bone trabeculae (arrowhead). Grade 4: Bone grafts with osteocytes and medullary cavity formation.

Dogs with OA score 2 showed moderate fibrosis of the joint capsule accompanied by more pronounced lymphoplasmacytic cellular infiltration. The articular cartilage showed initial signs of fibrocartilaginous metaplasia, fine fissures and incipient pannus formation. Early stages of osteophyte formation were identified, accompanied by an increased presence of chondroblasts, chondrocytes, osteoblasts and osteoclasts, indicating active remodeling processes. Subchondral bone areas showed mature bone layers confirming the onset of structural bone remodeling (Fig. 1). Radiographically this stage was defined by the presence of definite, but still small osteophytes, and sometimes even sclerosis could be seen.

### OA grade 3

Dogs with OA score 3 showed marked fibrosis of the joint capsule, characterized by increased collagen remodeling and intense lymphoplasmacytic cellular infiltration. The articular cartilage showed moderate to severe fibrocartilaginous metaplasia, structural disorganization and transmural damage. Pannus formation was clearly advanced, accompanied by subchondral osteoid trabeculae and medullary structures indicating active bone remodeling processes (Fig. 1). Radiographic findings at this stage included multiple moderate osteophytes in combination with sclerosis and developing bony deformities.

### OA grade 4

In animals with the highest severity (OA score 4), early signs of ankylosis were noted, including the formation of bone bridges. These changes were associated with an increased presence of osteocytes and the development of subchondral medullary spaces, reflecting advanced bone healing and adaptation processes (Fig. 1). Radiographically at this stage there were large osteophytes, definite sclerosis and bony deformity, osteoarthritis was categorized as grade 4.

### Synovial biomarkers: IL-6, TGF-β, osteocalcin and sRANKL

The analysis of biomarkers showed significant changes in cytokine and bone metabolism markers.

### Control group

Synovial fluid aspirates from animals without CCLR or knee osteoarthritis had no detectable concentrations of IL-6, TGF-β, osteocalcin or sRANKL.

### IL-6

**Fig. 2:**
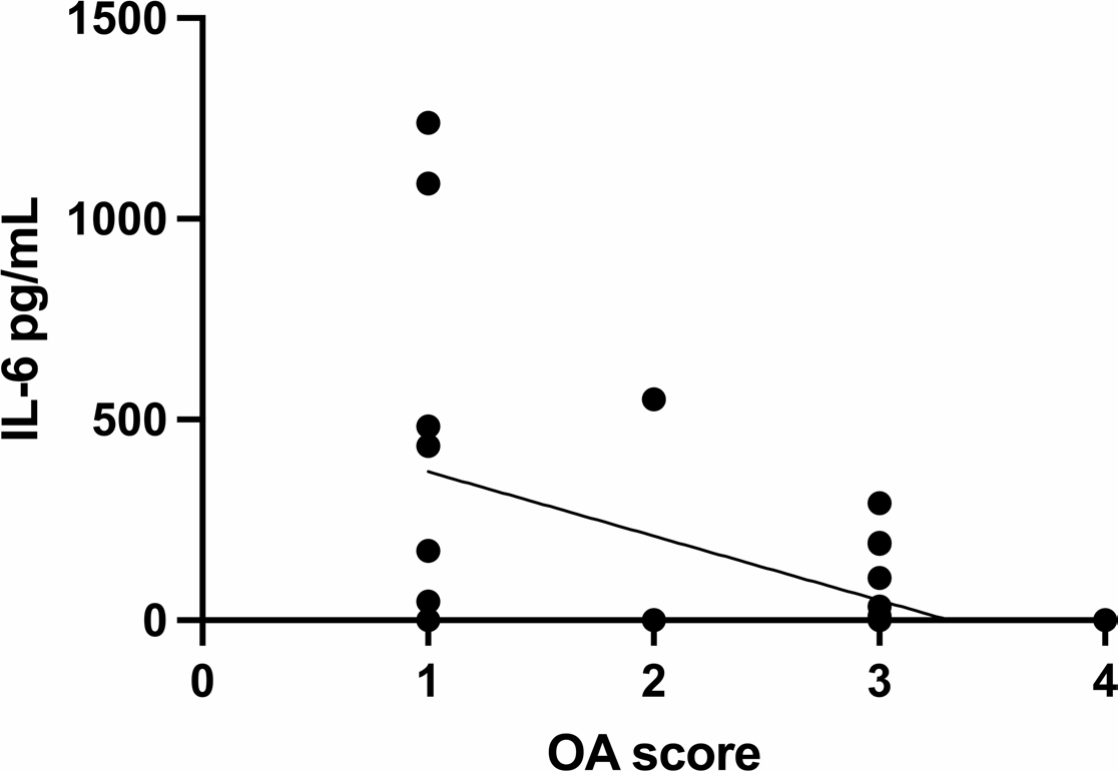
Concentration of IL-6 in the synovial fluid (SF) of canine knee joints with different stages of osteoarthritis, categorized according to the radiological scoring system.

Synovial fluid aspirates from 30 animals with different OA scores showed a negative correlation between IL-6 concentrations and OA severity (*r* = −0.2339; *p* = 0.0068). The correlation coefficient reflects a modest but statistically significant monotonic association between OA grade and IL-6 concentrations. While IL-6 was elevated in animals with mild changes (OA score 1), the concentration decreased as the disease progressed, indicating a reduction in inflammatory activity in favor of repair processes (Fig. 2).

**Fig. 3:**
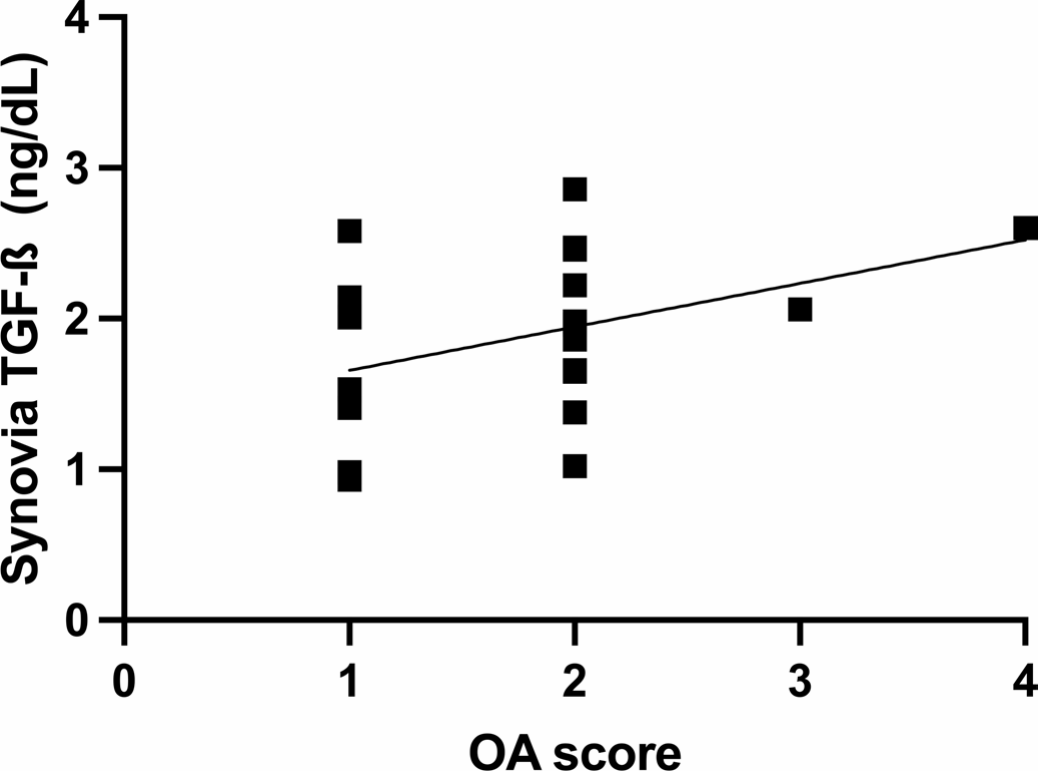
Concentration of TGF-β in the synovial fluid (SF) of canine knee joints with different stages of osteoarthritis, categorized according to the radiological scoring system.

### TGF-β

TGF-β levels, measured in 20 animals, showed no statistically significant association with OA severity (*r* = 0.154; *p* = 0.086). However, a non-significant trend towards higher TGF-β levels in advanced OA suggests a role for this cytokine in osteogenic processes (Fig. 3).

### Osteocalcin

**Fig. 4:**
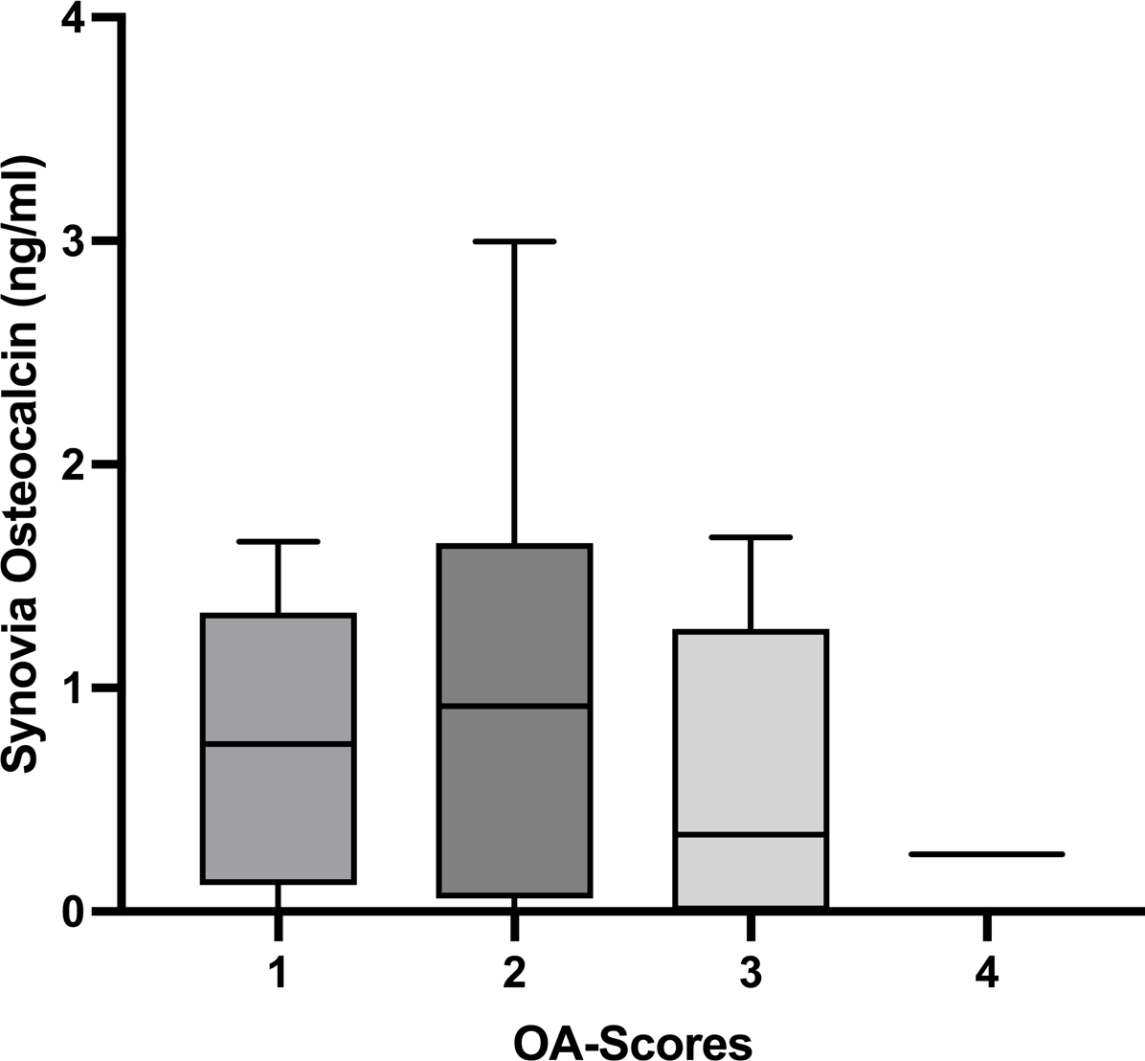
Concentration of canine osteocalcin in the synovial fluid (SF) of canine knee joints with different stages of osteoarthritis, categorized according to the radiological scoring system.

Osteocalcin concentrations were analyzed in 50 animals. A significant difference between early (OA score 1–2) and advanced (OA score 3–4) stages could not be detected. Nevertheless, the data showed a slight, non-significant decrease in osteocalcin levels as the disease progressed, possibly reflecting a change in bone remodeling activity (Fig. 4).

**Fig. 5:**
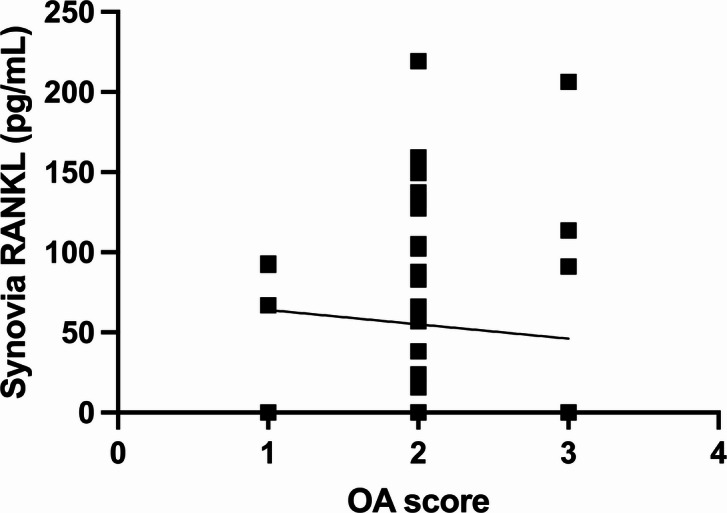
Concentration of sRANKL in the synovial fluid (SF) of canine knee joints with different stages of osteoarthritis, categorized according to the radiological scoring system.

### sRANKL

The concentration of sRANKL, a mediator of bone resorption, was measured in 28 dogs. The concentration was highest in OA score 2 animals. However, a regression analysis showed no significant correlation with OA scores (*r* = 0.03; *p* = 0.20). These results support the hypothesis that bone resorption is most active at intermediate OA stages (Fig. 5).

## Summary of the findings

The combination of histopathological changes and biomarker profiles emphasizes the role of OA as a dynamic interplay of inflammation, resorption and repair processes. While IL-6 indicates a transition from acute inflammatory to structural repair processes, the TGF-β trend reflects the promotion of osteogenic activities. The osteocalcin and sRANKL profiles emphasize the importance of bone remodeling processes in different OA phases.

These findings highlight that the balance between inflammatory and osteogenic mechanisms plays a key role in the pathogenesis and progression of OA.

## Discussion

Bone healing and pseudarthrosis are two processes of bone repair and remodeling that are controlled by cellular signaling, cytokine activity, and morphological changes and represent opportunities for bone repair depending on the initial situation. Our hypothesis is that osteoarthritis (OA) follows a bone healing response that is consistent with the physiologic stages observed in fracture repair. This model provides a conceptual framework for understanding the pathophysiologic mechanisms underlying OA as a chronic but adaptive response to joint loading and degeneration^23,24^.

### Mechanistic comparison of bone healing and OA pathophysiology

Bone healing usually proceeds in four different phases: Inflammation, soft callus formation, hard callus formation, and remodeling^23–25^. Similarly, OA progresses through comparable phases defined by cytokine signaling, cellular responses, and morphologic transitions.

Inflammation as the initial phase of bone healing involves the activation of cytokines such as TNF-α, IL-1 and IL-6^56,58,59^. These cytokines activate macrophages and initiate debris clearance while releasing growth factors that are critical for healing. Histopathologic observations in animals with OA score 1 in this study revealed inflammatory synovitis, lymphoplasmacytic cell infiltration, and synovial hyperplasia^60,61^, which are similar to the inflammatory phase observed in fracture healing.

The soft callus phase is characterized by the recruitment of mesenchymal stem cells (MSC) and their differentiation into chondrocytes, a process facilitated by TGF-β that regulates the synthesis of the extracellular cartilage matrix (ECM) and the migration of MSC^62–64^. The observations of fibro-cartilaginous metaplasia and pannus formation in dogs with OA score 2 in our study group are consistent with this phase. These morphologic changes signal chondrocyte activity and MSC recruitment to repair damaged articular cartilage and reflect the formation of soft callus during fracture repair^63^.

The formation of hard callus follows when osteoblasts differentiate from MSCs and begin to produce mineralized bone matrix, a process characterized by the activity of osteocalcin, a non-collagenous bone matrix protein essential for mineralization^65–67^. The findings of the examined dogs with OA score 3 showed increased formation of osteophytes, osteoid trabeculae and bone marrow, which corresponds to the hard callus phase. These findings support the concept that advanced OA reflects this stage of hard callus repair^67^.

Finally, the remodeling phase involves the coordinated activity of osteoclasts and osteoblasts controlled by the RANK/sRANKL/OPG signaling system, which maintains bone homeostasis through a balance between resorption and new bone formation^57,61,68^. In this study at OA score 4, histological evidence of ankylosis - characterized by bone bridging, osteocyte formation, and development of bone marrow cavities - reflects this remodeling phase, which is a structural response to chronic loading and repair^61^.

These observations collectively suggest that the progression of OA bears remarkable similarities to the physiologic phases of bone healing, supporting the hypothesis that OA represents a chronic but adaptive bone healing response^65^.

However, it must be acknowledged that synovial membrane pathology is a dynamic and heterogeneous process that does not necessarily progress in a strictly linear manner alongside structural changes to the subchondral bone. Unlike radiographic alterations, which typically reflect cumulative structural remodelling, synovial inflammation can fluctuate depending on disease duration, mechanical instability and individual immune responses.

For this reason, the OA scoring system in the present study prioritised structural radiographic progression as the main criterion for staging, while interpreting synovial findings as complementary rather than defining parameters. This approach reflects the staging of clinical disease severity rather than strict biological linearity in all joint compartments.

The cytokines and bone-related biomarkers analyzed in this study - IL-6, TGF-β, osteocalcin and sRANKL - support our hypothesis by revealing key phases of bone healing processes that correspond to the pathophysiology of OA.

IL-6 is an important cytokine involved in both inflammatory and anti-inflammatory processes. Our results show that IL-6 levels correlate negatively with the severity of OA (*p* = 0.0068, *r* = −0.2339). This suggests that as OA progresses, the inflammatory response is suppressed and shifts from acute inflammation to repair and bone remodeling processes^58,60,69^.

TGF-β serves as a chemotactic agent that facilitates MSC migration and differentiation during bone repair^59,62,63^. Although TGF-β levels increased with advancing OA stage, the correlation was not statistically significant. This result is consistent with previous observations showing that excessive TGF-β activation may impair joint function by promoting osteophyte formation^59,66^.

Elevated osteocalcin levels observed at OA score 3 indicate increased osteoblast activity and bone mineralization, consistent with the hard callus phase of bone healing^65–67^. This suggests that osteoblast-mediated bone remodeling is a hallmark of advanced stages of OA^61^.

Levels of sRANKL, which regulate osteoclast activity, were highest at OA score 2, supporting the hypothesis that bone resorption processes are critical during the soft callus phase. This emphasizes that dysregulated bone resorption is a contributing factor to the progression of OA^65,69^.

These cytokine and biomarker patterns are consistent with mechanisms known from bone healing processes, which further supports our hypothesis^61^. However, the results of our studies also show that the processes do not pass through the different stages statically, but dynamically, which explains the partial lack of significant correlations.

Stage-dependent trends were observed for TGF-β, osteocalcin and sRANKL; however, these associations did not reach statistical significance. Therefore, their potential role in canine osteoarthritis (OA) pathophysiology should be interpreted with caution.

Several factors may explain this lack of significance. Firstly, variability in disease duration prior to surgical intervention may have resulted in heterogeneous biomarker expression across animals within the same OA score. Secondly, the cross-sectional design of the study only captures single time points, rather than dynamic temporal changes, despite the fact that bone healing and OA progression are highly time-dependent processes. Thirdly, sample sizes differed between OA grades, particularly in advanced stages, which may have limited statistical power.

Despite the absence of statistical significance, the observed biological trends are consistent with the known mechanisms of bone remodelling and fracture healing. Therefore, while these findings cannot prove cause and effect, they do support the hypothesis that inflammatory and osteogenic pathways may interact during OA progression in dogs.

Several limitations of this study must be acknowledged.

Firstly, the cross-sectional study design allows identification of associations between biomarker expression and OA severity, but does not permit conclusions regarding temporal dynamics or causal relationships. Consequently, the observed correlations should be interpreted as descriptive rather than mechanistic evidence of disease progression. Secondly, the sample sizes were not evenly distributed across OA grades, with fewer cases in the more advanced stages. This imbalance may have reduced statistical power, contributing to the lack of significant correlations for certain biomarkers despite biologically plausible trends.

Thirdly, synovial fluid biomarkers reflect local joint conditions at a single time point and may not fully represent systemic bone remodelling processes or longitudinal changes occurring throughout disease progression. Additionally, variability in disease duration prior to surgical intervention may have introduced heterogeneity within OA grades.

Finally, the study focused on selected cytokines and bone-related markers associated with inflammation and remodelling. However, osteoarthritis is a complex, multifactorial disease involving additional molecular pathways that were not assessed in the present investigation.

Taken together, these limitations highlight the exploratory nature of the study and emphasise the need for larger, longitudinal and mechanistic investigations to further validate the hypothesis that OA is a dysregulated bone healing response.

## Conclusion

The findings of this study suggest that the morphological changes and selected biomarker patterns observed in canine osteoarthritis are similar to processes known to occur during fracture healing. In particular, stage-dependent alterations in inflammatory and bone-related mediators are consistent with inflammatory activation, tissue remodelling and structural adaptation.

Although not all biomarkers reached statistical significance, the combined morphological and biochemical observations support the hypothesis that osteoarthritis (OA) may involve mechanisms comparable to a dysregulated bone healing response, rather than representing a purely degenerative condition.

These data contribute to the growing body of evidence indicating that osteoarthritis involves dynamic interactions between inflammation, resorption, and bone formation. However, larger, longitudinal studies are required to further clarify causal relationships and determine the extent to which bone healing pathways contribute to OA progression.

## Methods

### Population of the study

The study cohort consisted of 111 dogs diagnosed with rupture of the cranial cruciate ligament (CCLR), all of which underwent surgery at the *Small Animal Clinic of the Veterinary Institute of the University of Göttingen*. The dog cohort represented a wide range of breeds, with mixed breeds predominating (*n* = 31). Of these 111 dogs, 45 were male, of which 11 were neutered, while 66 were female, of which 26 were neutered.

The average body weight was 23.9 kg, and the age of the dogs ranged from 1 to 13 years, with an average age of 6.4 years. In addition to the osteoarthritis (OA) cohort, synovial fluid samples were taken from 10 control dogs. These animals showed no clinical, radiographic or macroscopic evidence of ruptured cranial cruciate ligaments, osteoarthritis or other joint pathologies. The control samples were obtained from dogs that were euthanised for reasons unrelated to orthopaedic or systemic inflammatory disease. Prior to sample collection, a clinical evaluation and review of the medical history confirmed the absence of joint disease or chronic inflammatory disorders.

Synovial fluid was collected immediately post-mortem under sterile conditions.

These criteria ensured that the control samples represented physiologically normal joint conditions.

All experimental methods were carried out in accordance with the guidelines of experimental studies. The protocols were approved by the Lower Saxony Animal Welfare Authority (LAVES Nds. Landesamt für Verbraucherschutz und Lebensmittelsicherheit, Stau 75, D-26122 Oldenburg, germany; https://www.laves.niedersachsen.de), with a letter of confirmation, clarifying that no animal testing was performed as part of this study. In addition all methods are reported in accordance with the ARRIVE guidelines.

### Development of the OA scoring system

A scoring system for osteoarthritis (OA) was developed to standardize the comparison of disease progression and to compare the results with previous research findings. This scoring system was derived from preoperative radiographs and visual assessments of the affected knees by the surgeon. A modified Kellgren-Lawrence (KL) radiographic scoring system was introduced^70^(Table 1) using preoperative craniocaudal and mediolateral radiographic projections. Radiographic imaging was performed using the Siemens Opti 150/30/50 HC-100 (Siemens AG, Munich, Germany) for radiographic image acquisition, the Optimax Film Processor (1170-1-0000; PROTEC Medizintechnik GmbH & Co. KG, Oberstenfeld, Germany) for processing of the films, and FUJIFILM Super HR-E Medical Radiographic Films (18 × 24 cm, FUJIFILM Corporation, Tokyo, Japan) for image recording.

**Table 1:** The table describes the radiological findings in the various OA scores based on a modified Kellgren-Lawrence (KL) X-ray scoring system.

Kellgren-Lawrence system		Modified system for dogs	
Grade 0	Absence of radiographic changes	Grade 0	Absence of radiographic changes
Grade 1	Doubtful joint space narrowing and possible osteophytic lipping	Grade 1	Slightly detectable, small osteophytes and/or roughness, no sclerosis, no bony deformity
Grade 2	Definite osteophytes and possible joint space narrowing	Grade 2	Definite, small to moderate osteophytes, possible sclerosis, no bony deformity
Grade 3	Multiple osteophytes, definite joint space narrowing, sclerosis, possible bony deformity	Grade 3	Multiple and moderate to big osteophytes, initiating sclerosis, possible initiating bony deformity
Grade 4	Large osteophytes, marked joint space narrowing, severe sclerosis and definite bony deformity	Grade 4	Multiple and large osteophytes, sclerosis, advanced bony deformity

Radiologic assessment focused on key anatomic markers of OA, including subchondral sclerosis of the tibial plateau, presence of osteophytes on the sesamoid bones, proximal and distal patella, femoral sulcus, and lateral and medial collateral ligaments. The modified KL scoring was structured as follows:

Grade 1 is characterized by a slight roughness of the subchondral bone and the presence of subtle osteophytes. These changes are minimal and represent the earliest stage of osteoarthritic progression. In grade 2, small osteophytes less than 3 mm in size are observed. There are also signs of early changes in the subchondral bone, indicating the initial development of more pronounced structural changes. Grade 3 is characterized by the presence of multiple medium-sized osteophytes. Marked subchondral sclerosis, significant fibrosis of the synovial tissue and clear signs of bone deformity are also characteristic of this stage. The most advanced stage of osteoarthritis (grade 4) includes large osteophytes greater than 3 mm in size, marked subchondral sclerosis and extensive fibrotic changes in the synovial capsule. At this stage, visible deformations of the affected bone structure can also be observed.

X-ray examinations formed the basis for the correlation between the severity of the disease and the radiological changes.

### Surgical interventions and clinical observations

All surgical procedures were performed by a single experienced surgeon to maintain uniformity and ensure consistency of observations. Observations during the surgical procedures included the assessment of:

- Osteophyte growth and its morphological characteristics,
- Changes in the color of the synovial membrane (e.g. redness indicating inflammation),
- Viscosity of the synovial fluid,
- Thickness of the synovial membrane, classified as normal, slightly thickened or significantly thickened,
- Erosion of the cartilage or visible structural damage,
- Meniscal abnormalities, including deformities and tears.

Intraoperative findings were considered complementary descriptors of joint pathology. They provided additional information regarding inflammatory activity and tissue remodeling but did not independently override radiographic grading. Importantly, synovial membrane pathology was interpreted as a dynamic and potentially fluctuating component of OA that does not necessarily progress linearly alongside structural bone changes. Therefore, in cases where discrepancies existed between synovial inflammatory appearance and radiographic severity (e.g., pronounced osteophyte formation with minimal synovitis), radiographic criteria were prioritized for OA staging.

### Acquisition and processing of histological samples

During surgery, representative samples of synovial membrane and joint capsule tissue were obtained from macroscopically affected areas using sterile surgical instruments. In selected cases, small osteochondral samples were collected from areas adjacent to osteophyte formation when clinically indicated.

The tissue samples were fixed in 4% buffered formalin for 24–48 h and then routinely embedded in paraffin wax. Sections (3–5 μm thick) were prepared and stained with haematoxylin and eosin (H&E) for morphological evaluation.

It is important to note that histopathological analysis (H&E staining) was performed for morphological characterisation and illustrative purposes, but was not used to determine the OA score.

Where discrepancies existed between the appearance of synovial inflammation and radiographic severity (e.g. marked osteophyte formation with minimal synovitis), radiographic criteria were considered the primary determinant of OA grade, reflecting structural disease progression.

### Marker

Synovial fluid aspirates were taken for the biomarker analysis. These samples were obtained during the surgical procedure and isolated by aspiration from blood-free synovial fluid.

Dogs that had been treated with non-steroidal anti-inflammatory drugs (NSAIDs) or corticosteroids in the previous four weeks were excluded from the study, as these drugs could interfere with the assessment of bone healing markers and inflammatory responses. In addition, dogs with conditions other than osteoarthritis (OA) were excluded based on clinical, laboratory and imaging examinations.

These criteria ensured that the study population consisted exclusively of dogs with OA, providing a targeted and reliable basis for analyzing the pathology and progression of the disease.

Synovial fluid samples were collected under sterile conditions and immediately placed on ice. Within 30 min of collection, the samples were centrifuged at 3,000 g for 10 min. The supernatant was divided into smaller portions and stored at − 80 °C until analysis.

The samples were analysed without enzymatic pre-treatment (e.g. hyaluronidase digestion). Prior to ELISA measurement, the frozen aliquots were thawed once at room temperature and gently mixed to ensure homogeneity. Repeated freeze–thaw cycles were avoided.

### Marker analysis

To assess bone healing and inflammation, markers from synovial fluid were analyzed using quantitative immunoassays, focusing on cytokines and growth factors that are critical for bone remodeling processes. These included IL-6, TGF-β, osteocalcin and sRANKL, which are established biomarkers associated with inflammation and bone resorption processes.

### ELISA method

The following markers were analyzed using ELISA tests (enzyme-linked immunosorbent assays):

1. IL-6: Quantified with the Quantikine Canine IL-6 Immunoassay (CA 6000; R&D Systems, Minneapolis, MN, USA).

- Intra-assay precision was assessed with CVs of 3.3%, 3.2% and 2.25%, with mean concentrations of 92.6 pg/ml, 256.0 pg/ml and 685.0 pg/ml, respectively.

2. TGF-β1: Quantified using a Quantikine TGF-β1 sandwich immunoassay (catalog number DB100B; R&D Systems, Minneapolis, MN, USA).

- The intra-assay variability was between 3.4% and 6.7%.
- The inter-assay variability was between 5.7% and 8.9%.

3. Osteocalcin: Quantified with the Osteocalcin ELISA kit (No. E0800179; BlueGene Biotech, Shanghai, China).

- The intra-assay variability was < 10%.
- The inter-assay coefficient of variation was < 12%.

4. sRANKL: Quantified with the sRANKL ELISA kit (No. E08R0028; BlueGene Biotech, Shanghai, China).

- The intra-assay variability was < 10%.
- The inter-assay coefficient of variation was < 12%.

The ELISA protocols were performed in strict accordance with the manufacturer’s instructions. In brief, all reagents were brought to room temperature before use. Then 100 µl of the standard or sample was added to the antibody-coated wells. Subsequently, 50 µl of the enzyme conjugate was added and the mixture was incubated at 37 °C for 1 h. The substrate reaction was catalyzed by horseradish peroxidase (HRP) and colorimetric detection was performed spectrophotometrically at 450 nm with a wavelength correction at 570 nm using a microplate reader from Tecan Group Ltd. of Männedorf, Switzerland.

Not all synovial fluid aspirates were available in sufficient volume to analyse all biomarkers. Therefore, the number of samples analysed varied between assays: IL-6 (*n* = 30); TGF-β (*n* = 20); osteocalcin (*n* = 50); sRANKL (*n* = 28).

### Statistical analysis

Statistical analyses were performed using GraphPad Prism 8 (GraphPad Software, Inc.). Data distribution was assessed using the Kolmogorov–Smirnov test. As biomarker concentrations were not consistently normally distributed, the data are reported as the median and interquartile range (IQR). Comparisons between early OA stages (grades 1–2) and advanced OA stages (grades 3–4) were performed using the Mann–Whitney U test. Associations between OA grade (an ordinal scale) and biomarker concentrations were evaluated using two-tailed Spearman’s rank correlation. For correlation analyses, exact two-tailed p values were calculated based on Spearman’s rank correlation coefficients as implemented in GraphPad Prism. Given the ordinal nature of the OA score and the non-normal distribution of biomarker concentrations, non-parametric testing was considered appropriate. The significance threshold was set at *p* < 0.05.

As the study is evaluating the associations between osteoarthritis (OA) severity and four synovial biomarkers, the adequacy of the sample size was assessed based on the ability to detect monotonic correlations using Fisher’s z-transformation (two-sided α = 0.05, power = 80%). The minimum detectable correlation coefficients were found to be approximately 0.59 for *n* = 20 (TGF-β), 0.51 for *n* = 28 (sRANKL), 0.49 for *n* = 30 (IL-6) and 0.39 for *n* = 50 (osteocalcin). Therefore, the study is sufficiently powered to detect moderate-to-large associations; however, smaller effect sizes may not reach statistical significance, particularly in smaller subsets of assays. Consequently, findings without statistical significance are interpreted cautiously and considered exploratory.

## Supplementary Information

Below is the link to the electronic supplementary material.

Supplementary Material 1

## Data Availability

All data generated or analysed during this study are included in this published article as a supplementary information file.
